# Is it possible to claim or refute sputum eosinophils ≥ 3% in asthmatics with sufficient accuracy using biomarkers?

**DOI:** 10.1186/s12931-017-0615-9

**Published:** 2017-07-03

**Authors:** Sophie F. Demarche, Florence N. Schleich, Virginie A. Paulus, Monique A. Henket, Thierry J. Van Hees, Renaud E. Louis

**Affiliations:** 1 Department of Respiratory Medicine, CHU Liege, GIGA I3 Research Group, University of Liege, Liege, Belgium; 2 Department of Clinical Pharmacy, Center for Interdisciplinary Research on Medicines, University of Liege, Liege, Belgium

**Keywords:** Asthma, Phenotype, Biomarkers, Sputum, Blood, Eosinophils, Nitric oxide, Immunoglobulin E

## Abstract

The concept of asthma inflammatory phenotypes has proved to be important in predicting response to inhaled corticosteroids. Induced sputum, which has been pivotal in the development of the concept of inflammatory phenotypes, is however not widely available. Several studies have proposed to use surrogate exhaled or blood biomarkers, like fractional exhaled nitric oxide (FENO), blood eosinophils and total serum immunoglobulin E (IgE). However, taken alone, each of these biomarkers has moderate accuracy to identify sputum eosinophilia. Here, we propose a new approach based on the likelihood ratio to study which thresholds of these biomarkers, taken alone or in combination, were able to rule in or rule out sputum eosinophils ≥3%. We showed in a large population of 869 asthmatics that combining FENO, blood eosinophils and total serum IgE could accurately predict sputum eosinophils ≥ or <3% in 58% of our population.

## Introduction

Asthma is a heterogeneous airway inflammatory disease. With the emergence of sputum analysis, the eosinophilic and non-eosinophilic phenotypes have been described, which were suggested to have different sensitivities to inhaled corticosteroids (ICS) [1]. It was indeed shown that eosinophilic asthmatics responded better to ICS in terms of symptoms, respiratory function and bronchial hyperresponsiveness than their non-eosinophilic counterparts [2, 3].

Measuring sputum eosinophils in clinical practice is not applicable to the general population because it is technically demanding and time-consuming. To overcome this issue, user-friendly biomarkers like fractional exhaled nitric oxide (FENO), blood eosinophils and total serum immunoglobulin E (IgE) have been suggested as surrogate markers of airway eosinophilic inflammation [4]. Numerous thresholds for these biomarkers have been proposed to identify eosinophilic asthmatics [4–8], which may be confusing for the clinician. Moreover, each of these biomarkers taken alone has variable and moderate accuracy [4], that may be enhanced by combining them (like combining FENO and blood eosinophils [5]). Despite these efforts, there is currently no clear recommendation applicable to clinicians in their office practice to help them identify the eosinophilic status of their patients using the aforementioned biomarkers.

The purpose of our study was to assess whether several pragmatic thresholds of FENO, blood eosinophils and total IgE, taken alone or combined, were able to rule in or rule out sputum eosinophils ≥3%. In order to answer this question, we have calculated the likelihood ratio (LR) for each corresponding threshold or combination of thresholds, on a large database of asthmatic patients. The LR is a measure of diagnostic accuracy combining both the sensitivity and specificity, which is particularly useful for the clinician, with values above 10 or below 0.1 allowing to rule in or rule out the “disease” (i.e. eosinophilic asthma in this study) with strong evidence [9].

## Methods

We conducted a retrospective cross-sectional study on 869 asthmatics of varied severity recruited from the University Asthma Clinic of Liege, Belgium. Patients were eligible for the study if they had a visit with a successful measure of sputum eosinophils, FENO, blood eosinophils and total serum IgE (all performed on the same day) between January 2005 and September 2016. The study was approved by the ethics committee of the University Hospital of Liege (Ref 2016/276).

Asthma was diagnosed based on typical symptoms (wheezing, breathlessness, chest tightness, cough) and at least one of the following criteria: an improvement of 12% and 200 mL in forced expiratory volume in one second (FEV_1_) following inhalation of 400 μg salbutamol or a provocative concentration of methacholine causing a 20% fall in FEV_1_ (PC20M) <16 mg/mL. Sputum induction and processing were performed as previously described, using the whole expectorate technique [10]. The eosinophilic phenotype was defined as a sputum eosinophil count ≥3% [11]. FENO was measured at a flow rate of 50 mL/s (NIOX, Aerocrine, Solna, Sweden). Blood samples of patients were analysed by the routine laboratory of the University Hospital of Liege.

**Fig. 1:**
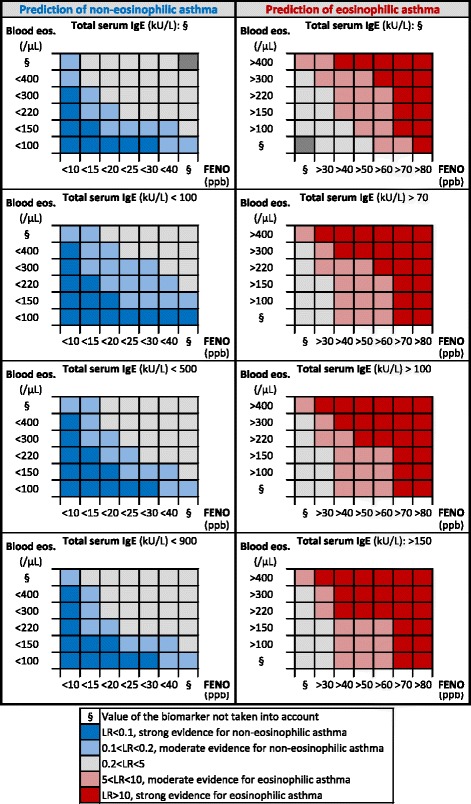
Representation of the strength of the likelihood ratio to predict non-eosinophilic (*left panel*) or eosinophilic (*right panel*) asthma according to several thresholds of FENO, blood eosinophils, total IgE or their combination. Each square of the Figure represents a subgroup of at least 20 patients. Abbreviations: *Blood eos* blood eosinophils, *FENO* fractional exhaled nitric oxide, *IgE* immunoglobulin E

Data were expressed as numbers and percentages for categorical variables and as median (first and third quartiles) or mean ± standard deviation for continuous variables. Non-eosinophilic asthmatics were compared to eosinophilic asthmatics using a Pearson’s chi-squared test for categorical variables, a Student’s t-test for parametric continuous variables, and a Mann-Whitney test for non-parametric continuous variables. To assess the ability of FENO, blood eosinophils, total IgE or their combination to rule in or rule out eosinophilic asthma, we chose pragmatic thresholds for each biomarker based on previously published data [4–8], with 2 additional thresholds to improve the practicality of the tool: 150 blood eosinophils/μL and 500 kU total IgE/L. These thresholds are presented in Fig. 1. The LR calculation was performed in two parts. In the first part (presented in the right side of Fig. 1), we calculated the positive LRs to assess which threshold(s) was (were) able to rule in eosinophilic asthma. For each threshold or combination of thresholds, the positive LR was calculated as follows: % of eosinophilic asthmatics whose value(s) of the biomarker(s) was (were) above the threshold(s)/% of non-eosinophilic asthmatics whose value(s) of the biomarker(s) was (were) above the threshold(s). This formula actually corresponds to: sensitivity/(1-specificity). Positive LR values above 10 allow to rule in eosinophilic asthma with strong evidence [9] and are highlighted by dark red in Fig. 1. In the second part (presented in the left side of Fig. 1), we calculated the negative LRs to assess which threshold(s) was (were) able to rule out eosinophilic asthma (i.e. to rule in non-eosinophilic asthma). For each threshold or combination of thresholds, the negative LR was calculated as follows: % of eosinophilic asthmatics whose value(s) of the biomarker(s) was (were) below the threshold(s)/% of non-eosinophilic asthmatics whose value(s) of the biomarker(s) was (were) below the threshold(s). This formula actually corresponds to: (1-sensitivity)/specificity. Negative LR values under 0.1 allow to rule out eosinophilic asthma (i.e. to rule in non-eosinophilic asthma) with strong evidence [9] and are highlighted by dark blue in Fig. 1. Positive LRs between 5 and 10, and negative LRs between 0.1 and 0.2 give moderate evidence [8] to rule in or rule out eosinophilic asthma, and are only given in Fig. 1 for information. To ensure the reliability of the results, only subgroups including at least 20 patients were considered for the LR calculation. A *p* value <0.05 was considered statistically significant. Statistical analysis was done using STATA version 13.0 (Statistical Software, College Station, TX: StataCorp LP).

## Results

Our cohort included 869 patients. Patient characteristics of the total population and comparison between non-eosinophilic and eosinophilic asthmatics are shown in Table 1. The prevalence of eosinophilic asthma was 43%. Figure 1 represents the strength of the LR for each threshold of FENO, blood eosinophils and total IgE, taken alone or in combination. LRs above 10 give strong evidence to rule in eosinophilic asthma and are highlighted by dark red in Fig. 1 while LRs below 0.1 give strong evidence to rule out eosinophilic asthma (i.e. to rule in non-eosinophilic asthma) and are highlighted by dark blue in Fig. 1 [9]. The LRs for a single biomarker can be found in Fig. 1 where the values of the other two biomarkers are not taken into account. Likewise, the LRs for combinations of two biomarkers are found in the grid where the value of the third biomarker is not taken into account. For example, a patient with <20 ppb of FENO has not a strong prediction of non-eosinophilic asthma (0.2 < LR < 5), while a patient with <20 ppb of FENO and <100 blood eosinophils/μL has a strong prediction of non-eosinophilic asthma (LR < 0.1).

**Table 1:** Patient characteristics

	Total population	Non-eosinophilic asthmatics (NEA)	Eosinophilic asthmatics (EA)	p (NEA vs EA)
N	869	495	374	-
Women, N (%)	505 (58)	314 (63)	191 (51)	<0.001
Age, years	49 (35–61)	46 (33–59)	51 (38–63)	<0.001
BMI, kg/m^2^	26.2 ± 5.2	26.3 ± 5.3	26.2 ± 5.0	0.96
Atopy, N (%)	482 (56)	251 (51)	231 (62)	0.001
Smoking status, N (%)				
Non-smokers	457 (53)	262 (53)	195 (52)	0.049
Current smokers	187 (21)	118 (24)	69 (18)	
Ex-smokers	225 (26)	115 (23)	110 (29)	
FEV_1_, % predicted	83.6 ± 20.6	86.6 ± 19.8	79.6 ± 20.9	<0.001
FEV_1_/FVC, %	72.6 ± 11.1	74.4 ± 10.6	70.1 ± 11.3	<0.001
PC20M, mg/mL^a^	2.9 (0.7–13.0)	3.7 (0.9–15.0)	1.8 (0.5–9.8)	<0.001
Reversibility, %	7 (2–12)	6 (2–11)	8 (3–14)	<0.001
ACQ score	2.0 ± 1.2	1.9 ± 1.1	2.2 ± 1.3	<0.001
AQLQ score	4.5 ± 1.4	4.5 ± 1.3	4.5 ± 1.4	0.38
FENO, ppb	23 (13–45)	17 (12–29)	41 (21–72)	<0.001
Sputum eosinophils, % of non-squamous cells	1.8 (0.2–10.8)	0.2 (0.0–1.0)	13.2 (6.5–35.0)	<0.001
Sputum neutrophils, % of non-squamous cells	53 (28–76)	64 (39–84)	41 (22–59)	<0.001
Total serum IgE, kU/L	121 (37–328)	79 (23–227)	200 (80–472)	<0.001
Blood eosinophils, cells/μL	188 (109–328)	137 (80–217)	290 (189–507)	<0.001
Blood neutrophils, cells/μL	4068 (3150–5402)	4034 (3058–5498)	4078 (3228–5312)	0.58
ICS category, N (%)				
Steroid naive	314 (36)	204 (41)	110 (30)	0.003
Low dose^b^	126 (15)	71 (14)	55 (15)	
Medium dose^b^	182 (21)	97 (20)	85 (23)	
High dose^b^	242 (28)	121 (25)	121 (33)	
OCS therapy, N (%)	62 (7)	30 (6)	32 (9)	0.16
LABA, N (%)	530 (61)	279 (56)	251 (67)	0.001
LTRA, N (%)	220 (25)	130 (26)	90 (24)	0.46
Theophylline, N (%)	25 (3)	17 (3)	8 (2)	0.26

^a^Data available for 493 patients of the total population: 312 non-eosinophilic patients and 181 eosinophilic patients

^b^Low dose ICS: ≤ 500 μg per day; medium dose ICS: >500–1000 μg per day; high dose ICS: >1000 μg per day beclomethasone dipropionate - chlorofluorocarbon

Abbreviations: *ACQ* asthma control questionnaire, *AQLQ* asthma quality of life Questionnaire, *BMI* body mass index, *FENO* fractional exhaled nitric oxide, *FEV*_*1*_ forced expiratory volume in 1 s, *FVC* forced vital capacity, *ICS* inhaled corticosteroid, *IgE* immunoglobulin E, *LABA* long-acting β2-agonist, *LTRA* leukotriene receptor antagonist, *OCS* oral corticosteroid, *PC20M* provocative concentration of methacholine causing a 20% fall in FEV_1_

If we focus on clinically relevant LRs for the biomarkers taken alone, a value of FENO >80 ppb was associated with a LR above 10 (Fig. 1). In our population, 10% (89/869) of patients had a FENO >80 ppb, of whom 79 (89%) were actually eosinophilic. Likewise, a value of blood eosinophils >1000 cells/μL was associated with a LR above 10. In our study, 2% (20/869) of patients had >1000 blood eosinophils/μL, of whom 18 (90%) were actually eosinophilic. This threshold was not shown in Fig. 1 because the subgroup it determined only included 20 patients and a combination with other biomarkers would necessarily lead to <20 patients per group. No threshold of FENO or blood eosinophils used as single biomarkers was associated with a LR below 0.1. As for total IgE, no threshold was able to reach a LR above 10 or below 0.1. When considering FENO, blood eosinophils or their combination without using total IgE (corresponding to the upper left panel and the upper right panel of Fig. 1), 45% of the population (393/869) was included in at least one category associated with a LR above 10 or below 0.1. Of these 393 patients, 352 (90%) were properly classified into eosinophilic or non-eosinophilic asthma. Finally, when considering FENO, blood eosinophils, total IgE or their combination (corresponding to the entire Fig. 1), 58% of our population (506/869) was included in at least one category associated with a LR above 10 or below 0.1. Of these 506 patients, 440 (87%) were properly classified into eosinophilic or non-eosinophilic asthma. The number of subjects with a strong evidence of eosinophilic or non-eosinophilic asthma was therefore significantly increased from 393 to 506 patients when IgE levels were taken into account (*p* < 0.0001 using the McNemar test), while keeping an accuracy of nearly 90%.

In asthmatics untreated with ICS (*N* = 314), 189 patients (60%) were included in at least one category with a LR above 10 or below 0.1 in Fig. 1, of whom 169 (89%) were correctly classified. In asthmatics treated with ICS (*N* = 555), 317 patients (57%) were included in at least one category with a LR above 10 or below 0.1, of whom 271 (85%) were correctly classified. The proportion of patients with a strong evidence of eosinophilic or non-eosinophilic asthma and the correctness of the tool were not statistically different between patients untreated and treated with ICS (*p* = 0.38 and *p* = 0.20, respectively).

## Discussion

In our study, we showed that FENO and blood eosinophils used as single biomarkers were able to accurately predict sputum eosinophils ≥3% in a very small proportion of patients (10% and 2%, respectively), while IgE levels taken alone were unable to accurately predict the eosinophilic status. Overall, these results are consistent with previous studies reporting that these biomarkers are not sufficiently good predictors of the airway eosinophilic status when used as single surrogate markers [4, 12].

Hastie et al. showed that combining FENO and blood eosinophils did not accurately predict the eosinophilic phenotype in asthmatic patients, with an accuracy (defined as the percentage of subjects correctly classified by the test as having or not having eosinophilic asthma) of 55% [12]. When using FENO and blood eosinophils, our data show that we can only achieve a satisfactory prediction in 45% of patients, which is not very different from what Hastie et al. reported. We also found that measuring total IgE may be of interest as the proportion of asthmatics accurately predicted rose from 45 to 58% of the population, which supports the idea that combining biomarkers is useful [13].

Our results highlight the fact that these three surrogate markers are not able to entirely replace the sputum analysis to determine the eosinophilic status of patients. However, we still believe that the use of these biomarkers is valuable in routine clinical practice. Their measure is easy to perform, well standardised, less costly than sputum analysis, and their results are rapidly available. Therefore, we may suggest measuring these biomarkers in asthmatic patients in whom the practitioner wants to know the eosinophilic phenotype. For the 58% of patients identified by the tool as having a strong evidence of eosinophilic or non-eosinophilic asthma, a sputum induction might be spared. However, for the remaining 42% of patients, a sputum induction would be recommended in a dedicated centre to identify their eosinophilic status.

One limitation of our study is its retrospective design. Moreover, the accuracy of the tool in Fig. 1 should also be studied in other centres performing sputum in asthmatic patients to assess its generalisability (external validity) [14]. Nevertheless, a strength of our study is that our asthmatic population encompassed the all spectrum of severity and our grid may therefore be applicable to a majority of patients seen in clinical practice. Although ICS are known to impact sputum and blood eosinophils as well as FENO [15], our data show that the tool is suitable for asthmatic patients regardless of treatment with ICS. Therefore, the Figure could be printed and kept in the pocket of the practitioner as a companion tool to profile the eosinophilic phenotype in asthma.

In conclusion, combining exhaled breath and blood biomarkers to identify airway eosinophilia appears to be a valuable attitude in clinical practice in around 60% of patients, leaving the remaining 40% with uncertain eosinophilic status, which should be investigated by a sputum analysis.

## Abbreviations

FENO: fractional exhaled nitric oxide
FEV_1_: forced expiratory volume in one second
ICS: inhaled corticosteroid
IgE: Immunoglobulin E
LR: likelihood ratio
PC20M: provocative concentration of methacholine causing a 20% fall in FEV_1_
